# Covalently Coupled Phenylenediamine and Citric Acid-Based Carbon Dots: A Dual-Emissive Architecture for Rapid Optical Cr(VI) Sensing and Live-Cell Imaging

**DOI:** 10.1021/acsomega.6c01832

**Published:** 2026-06-25

**Authors:** Sultan Şahin Keskin, Canan Çil, Abdulhalim Kılıç, Levent Trabzon, Caner Ünlü

**Affiliations:** a Department of Genetic and Bioengineering, Alanya Alaaddin Keykubat University, Antalya/Alanya 07425, Turkey; b MEMS Research Center, 52971 Istanbul Technical University, Istanbul 34469, Turkey; c Department of Molecular Biology and Genetics, Faculty of Science and Letters, 52971 Istanbul Technical University, Istanbul 34469, Turkey; d Molecular Biology-Genetics and Biotechnology Program, MOBGAM, 52971 Istanbul Technical University, Istanbul 34469, Turkey; e Department of Mecanical Engineering, Faculty of Mechanical Engineering, 52971 Istanbul Technical University, Istanbul 34437, Turkey; f Department of Chemistry, Faculty of Science and Letters, 52971 Istanbul Technical University, Istanbul 34469, Turkey

## Abstract

Carbon dots (CDs) are fluorescent nanomaterials known for their excellent photostability, tunable fluorescence, and biocompatibility, making them ideal for various applications. Within this class, dual-emissive CDs are particularly noteworthy, as they can exhibit two distinct emission peaks under a single excitation wavelength. This study reports the development of covalently coupled dual-emissive CDs, synthesized from 1,2-phenylenediamine and citric acid, which exhibit distinct dual emission peaks at 455 and 555 nm under a single 390 nm excitation wavelength. These coupled CDs served as a selective fluorescent probe for Cr­(VI) detection, demonstrating a linear response of the emission peak at 455 nm across the range of 5 to 500 μM. The quenching mechanism was investigated through time-resolved photoluminescence (TRPL) and spectral overlap analysis. The results indicated that the fluorescence response was predominantly structured by the inner filter effect, while the nearly unchanged fluorescence lifetime suggests that dynamic collisional quenching is not the dominant pathway. A key advantage of this system is its rapid Cr­(VI) detection capability, with a response time of less than 15 s. Furthermore, the probe was successfully applied for visualizing Cr­(VI) detection within NIH/3T3 cells through fluorescence imaging, and the probe showed low cytotoxicity under the short-term imaging conditions, with cell viability remaining above 90% after 4 h exposure at concentrations up to 5 mg/mL. The covalent coupling strategy yields a stable dual-emissive material with enhanced sensing performance for Cr­(VI) compared to individual or simply mixed CDs, highlighting its potential for environmental monitoring and bioimaging applications.

## Graphical abstract

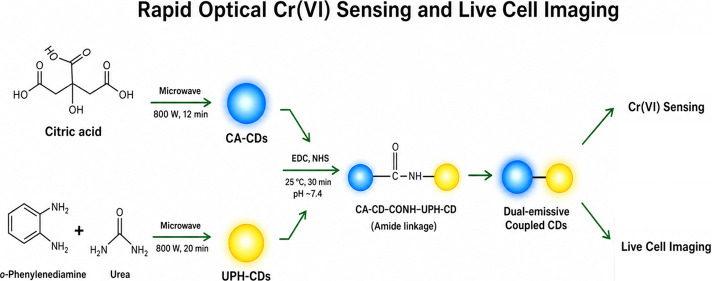

## 1 Introduction

Carbon-based fluorescent materials have garnered significant attention in various fields among fluorescence-based sensing materials owing to their high efficiency, tunable photoluminescence (PL), biocompatibility, and strong photostability in the visible region. Their facile synthesis and low toxicity further enhance their appeal over traditional organic dyes. Within this class, dual-emissive carbon dots (CDs) are particularly noteworthy for their ability to exhibit two distinct emission peaks under a single excitation wavelength. In recent years, white light emitters derived from dual-emissive CDs have been developed for diverse applications, including anticounterfeiting via multicolor fluorescence codes,^1,2^ solid-state illumination for white light-emitting diodes,^3,4^ and sensing with ratiometric fluorescent probes.^5–11^ Additionally, dual-emissive CDs are increasingly utilized in *in vivo* and *in vitro* multicolor imaging due to their biocompatibility and the ease of functionalizing their surface groups for biological systems.^12–14^

Recent advancements highlight the versatility of CDs and their precursors in diverse sensor and materials science applications.^15^ For instance, Yuan et al. developed dual-signal uric acid sensing platforms based on carbon quantum dots and *o*-phenylenediamine.^16^ Concurrently, citric acid-based CDs are being actively explored for energy conversion technologies, as reviewed by Vallan and Imahori.^17^ In a different approach, Arkin et al. prepared polychromatic carbon dots from *m*-phenylenediamine and urea, demonstrating their utility as multifunctional fluorescent probes.^18^ More recently, Heng et al. reported a dual-mode ratiometric fluorometric and colorimetric platform using nitrogen-doped CDs and *o*-phenylenediamine for nitrite detection.^19^ Existing dual-emissive CDs often rely on single-precursor synthesis, which limits the ability to independently tune the emission wavelengths and quantum yields of the emissive centers. While physical mixing of two different CDs is a simpler alternative, it frequently results in instability and uncontrolled quenching because of electrostatic interactions between the nanoparticles. Covalent postcoupling via carbodiimide chemistry addresses these challenges by creating a stable, rigid framework that prevents self-quenching while maintaining the distinct optical signatures of both precursors. This approach not only ensures structural integrity but also facilitates rapid analyte interaction, leading to a superior response time and sensitivity compared with traditional architectures.

Chromium (Cr) is a widespread metallic element; however, its hexavalent form, Cr­(VI), is a recognized environmental contaminant and human carcinogen due to its high oxidation potential and ability to interact with DNA.^20–22^ This toxicity necessitates sensitive and rapid methods for Cr­(VI) detection. While traditional techniques like atomic absorption spectrometry and inductively coupled plasma mass spectrometry are effective, they often require costly equipment and complex, time-consuming procedures.^23–25^ Fluorescence-based sensing, particularly using CDs, offers a promising alternative due to its operational simplicity, cost-effectiveness, rapid response, and potential for visual detection.^26–30^ In recent years, studies in optical Cr­(VI) sensing by carbon dots have tended to increase.^31–35^ For instance, N-doped citric acid-based dual-emissive CDs achieved a detection range of 25 μM to 1 mM and a limit of detection (LOD) of 3.2 μM.^7^ Similarly, Campos et al.^36^ utilized silicon quantum dots coated with hydroxyl poly­(amidoamine) dendrimers to detect Cr­(VI), reporting a linear range of 5–288 μM and an LOD of 2.17 μM. Furthermore, B–N codoped dual-emissive CDs provided a linear range of 0–100 μM with an LOD of 0.41 μM,^10^ while CDs derived from *Lycium barbarum* demonstrated a detection range of 0–40 μM with an LOD of 0.16 μM and a response time of 1 min.^31^

While these studies underscore the broad utility of CD-based materials, the present work establishes its novelty through a specific focus on the covalent coupling of two distinct, presynthesized CD populations: citric acid-derived CA-CDs and 1,2-phenylenediamine/urea-derived UPH-CDs. This approach differs significantly from those utilizing single-type polychromatic CDs or systems where 1,2-phenylenediamine may serve as a coreactant within the sensing mechanism. Our methodology emphasizes the formation of stable amide bonds to link these disparate CD types, creating a robust, dual-emissive architecture. This engineered coupled system is applied as a selective fluorescent probe for rapid Cr­(VI) detection, achieving a response time of less than 15 s. This specific application in rapid Cr­(VI) sensing, combined with the unique covalent coupling strategy, distinguishes our work from the broader applications in energy conversion or the detection of other analytes, such as uric acid or nitrite, described in the aforementioned literature. Furthermore, this study provides a systematic comparison of the optical and sensing performance of these covalently coupled CDs against their simply mixed counterparts, thereby highlighting the distinct advantages conferred by the covalent linkage for achieving stable dual emission and effective analyte detection. Double-emissive CDs also offer significant advantages in environmental-sensing applications due to their ability to provide dual fluorescence signals. Their excellent photostability ensures long-term durability under challenging environmental conditions.

Therefore, this study details the synthesis of dual-emissive CDs by covalently linking citric acid-based CDs (CA-CDs) with *o*-phenylenediamine-based CDs (UPH-CDs) using carbodiimide chemistry. We investigated the optical properties of these coupled CDs, highlighting their white light emission resulting from dual emission peaks. The synthesized probe was systematically evaluated for its selectivity and sensitivity in Cr­(VI) detection, demonstrating a significant quenching of the CA-CD emission peak (455 nm) while the UPH-CD emission (555 nm) was less affected, demonstrating a linear response across the range of 5 to 500 μM. Finally, the applicability of these coupled CDs was confirmed through live-cell imaging in NIH/3T3 cells, alongside cytotoxicity assessments.

## 2 Materials and Methods

### 2.1 Materials

1-Ethyl-3-(3-(dimethylamino)­propyl) carbodiimide (EDC), *N*-hydroxysuccinimide (NHS), Fe­(II) chloride tetrahydrate, Fe­(III) chloride hexahydrate, Co­(II) sulfate heptahydrate, zinc chloride, silver nitrate, and nickel­(II) sulfate hexahydrate were purchased from Sigma-Aldrich Co. (St. Louis, MO, USA). Potassium chromate, cadmium acetate, Cu­(II) chloride dihydrate, manganese­(II) chloride dihydrate, potassium chromate, chromium­(III) nitrate nonahydrate, *o*-phenylenediamine, urea, and citric acid were purchased from Merck (Darmstadt, Germany). The dialysis membrane (Spectra/Por Biotech, MWCO: 500–1000 Da) was purchased from Repligen Corporation. For cell culture studies, Dulbecco’s modified Eagle’s medium (DMEM), fetal bovine serum (FBS), penicillin-streptomycin, and trypsin/EDTA were obtained from PAN Biotech (Aidenbach, Germany). Cell counting kit-8 (CCK-8) was obtained from Dojindo Molecular Technologies, Inc. (Kumamoto, Japan).

### 2.2 Methods

A traditional microwave-assisted method^37,38^ was applied to synthesize CA-CDs and UPH-CDs.

#### Synthesis of CA-CDs

For the synthesis of CA-CDs, 1 g of citric acid as the carbon precursor was placed into a kitchen-type microwave oven (SAMSUNG/model no. MS23F300EES) and heated at 800 W on a rotating plate for 12 min. The synthesis was carried out in a 50 mL flat-bottom flask, ensuring uniform exposure to the 800 W microwave irradiation. At the end of the synthesis procedure, solid products with a brownish color were obtained. Then, the solid products were dispersed in 10 mL of ultrapure water, and the first big clusters were removed by a filter paper with a 10 μm pore size and then filtered through a syringe filter with a 0.22 μm pore size. In the last step, the solvents of the purified CD aqueous dispersion were dried and the purified solid product was kept at room temperature in a dark place for further analysis. These CA-CDs primarily provide the blue-emissive component centered at approximately 455 nm.

#### Synthesis of UPH-CDs

For the synthesis of UPH-CDs, 400 mg of *o*-phenylenediamine and 100 mg of urea in 20 mL of ultrapure water were placed into a kitchen-type microwave oven and heated at 800 W on a rotating plate for 20 min. The synthesis was carried out in a 50 mL flat-bottom flask, ensuring uniform exposure to the 800 W microwave irradiation. At the end of the synthesis procedure, solid products with a brownish color were obtained. Then, the solid products were dispersed in 10 mL of ultrapure water, and the first big clusters were removed by a filter paper with a 10 μm pore size and then filtered through a syringe filter with a 0.22 μm pore size. In the last step, the solvents of the purified CD aqueous dispersion were dried and the purified solid product was kept at room temperature in a dark place for further analysis. The use of urea in UPH-CDs synthesis allows for the doping of nitrogen (pyridinic/pyrrolic N) to the core of CD, resulting in surface passivation through the amine groups on the surface, which increases the quantum yield of the resulting CD.^37^ UPH-CDs exhibiting a yellow emission centered at approximately 555 nm were obtained.

#### Synthesis of Coupled CDs

The covalently coupled dual-emissive CDs were prepared via a postsynthetic coupling strategy using carbodiimide chemistry to link the carboxyl groups of presynthesized CA-CDs with the amine groups of presynthesized UPH-CDs. Specifically, carboxyl groups on the surface of CA-CDs were activated using carbodiimide chemistry followed by a reaction with amino groups present on UPH-CDs to form amide bonds. This approach enables the covalent linkage between two distinct emissive carbon dot populations rather than generating a single-population dual-emissive system. Specifically, 250 mM EDC (1-ethyl-3-(3-(dimethylamino)­propyl) carbodiimide) was added to 8 mL of 7.5 mg/mL CA-CDs in water and left shaken for 10 min. Subsequently, 300 mM NHS (*N*-hydroxysuccinimide) was added to the mixture and shaken for another 15 min at room temperature. To remove unreacted EDC and NHS, dialysis (MWCO: 500–1000 Da) was performed for 2 h, with the pure water replenished every 30 min. The carboxyl-activated CA-CDs were then mixed with UPH-CDs (5 mg/mL), which possess amine groups, in PBS (pH 7.45) and allowed to react overnight with shaking. For comparison, a physical mixture of CA-CDs and UPH-CDs was prepared by using identical concentrations without the coupling reagents. This covalent coupling strategy yields a stable dual-emissive nanostructure that preserves the intrinsic emission characteristics of both precursors while preventing aggregation and uncontrolled energy transfer typically observed in physically mixed systems.

#### Characterization of Coupled Carbon Dots

To obtain detailed information about the bonding, coupled and mixed CDs were characterized by attenuated total reflectance Fourier transform infrared (ATR-FTIR) spectroscopy. The FTIR spectra of CDs were measured in the range of 400–4000 cm^–1^ by using a Bruker ATR-FTIR spectrophotometer (Bruker Co. USA). The zeta potential of CDs was determined with a Malvern Zetasizer (Malvern Panalytical, Malvern, UK). Dynamic light scattering (DLS) was peformed using the same Malvern Zetasizer on aqueous dispersions of the nanoparticles. The morphology and size of CDs were characterized by transmission electron microscopy (TEM).

Optical characterizations of CA-CDs, UPH-CDs, coupled CDs, and simply mixed CDs to compare each other were studied by fluorescence and UV–vis spectroscopy. The absorption spectra of CDs were recorded by an N4S/N4 UV–visible spectrophotometer. The emission spectra of CDs were recorded by a Varian Cary Eclipse fluorescence spectrofluorometer (Varian Inc., Palo Alto, CA, USA) using various excitation wavelengths (λ_exc_) between 320 and 480 nm. The emission color of each sample was examined under 366 nm UV-light irradiation. The photostabilities of the coupled CDs were checked by recording the change in intensity of the emission at 455 nm upon continuous illumination with 380 nm (100 mW/cm^–2^) in a 3 mL quartz cuvette under constant stirring at 25 °C for 1 h. All of the optical characterizations were performed on aqueous dispersion, and all spectroscopic analyses were conducted at room temperature. Moreover, the stability of CDs within 60 min and 20 days was investigated.

Quantum yields of CDs and coupled CDs were determined at an excitation wavelength of 380 nm by eq 1;^39^

$$\Phi_{x}=\Phi_{\mathrm{st}}\frac{I_{x}}{I_{\mathrm{st}}}\frac{A_{\mathrm{st}}}{A_{x}}\frac{{\eta_{x}}^{2}}{{\eta_{\mathrm{st}}}^{2}}$$ 1

where Φ is the quantum yield, *I* is the integrated fluorescence intensity, *A* is the absorbance at excitation wavelength, and η is the refractive index of the solvent. The subscripts st and x refer to standard with known quantum yield and the sample, respectively. Coumarin 102 in ethanol (Φ = 0.93) was chosen as a standard.

#### Cr­(VI) Sensing

The emission spectrum of coupled CDs (1:1 v:v with the analyte solution: 1 mL of 9 mg/mL CD solution mixed with 1 mL of the Cr­(VI) sample) in the presence of various concentrations of Cr­(VI) was recorded with excitation at 380 and 410 nm, presumably using the Varian Cary Eclipse fluorescence spectrofluorometer. To evaluate the selectivity, other metal ions (Cr^3+^, Mn^2+^, Co^2+^, Cu^2+^, Fe^2+^, Fe^3+^, Hg^2+^, Cd^2+^, Ni^2+^, Ag^+^, and Zn^2+^) at a concentration of 500 μM were added into coupled CDs (9 mg/mL) (1:1 v:v), and their corresponding fluorescence emission spectra were recorded with excitation at 380 nm.

For real-sample analysis, tap water was collected from a laboratory source. Experiments were conducted by adding known concentrations of Cr­(VI) (7.8, 31.25, 125, and 500 μM) to the tap water samples. The fluorescence of the coupled CD probe was then measured upon addition to these samples.

#### Cell Culture

The NIH/3T3 mouse fibroblast cell line was cultured regularly using DMEM supplemented with 10% (v/v) FBS in a humidified incubator with 5% CO_2_ at 37 °C. The cells were detached from the culture flask using trypsin–EDTA solution when they reached confluence. They were transferred into a 48-well plate (5 × 10^4^ cells/well) for fluorescence imaging and a 96-well plate (1 × 10^4^ cells/well) for the cytotoxicity assays. Then, the cells were preincubated for 24 h in the incubator.

#### Cytotoxicity Assay

For the cytotoxicity studies, the coupled CD stock solution in PBS pH 7.4 was complemented with DMEM (w/o phenol, w/o FBS) at a 1:5 (v/v) ratio. The cells were treated with various concentrations of the probe (0, 0.1, 1, 2, and 5 mg/mL) for 4 h. After treatment, cells were washed with PBS and incubated with the CCK-8 kit for 2 h. Then, the medium of each well was transferred to others, and the absorbance was measured at 450 nm. Cell viability was calculated for each group, with the values of the control group containing cells that were not treated with the probe as 100%.

#### Fluorescence Imaging

For fluorescence imaging, NIH/3T3 cells were treated with a 2 mg/mL probe solution in DMEM (without FBS) for 1 h in the incubator (37 °C, 5% CO_2_). Subsequently, 50 and 250 μM Cr­(VI) (prepared from potassium chromate in PBS) were added to the cells, and the cells were incubated for an additional 2 h at 37 °C. Following both incubations, the cells were rinsed with PBS to remove any residual probe and Cr­(VI) that had not entered the cells. Lastly, the cells were observed under an inverted fluorescence microscope (Axiovert A1, Zeiss, Oberkochen, Germany) using a DAPI filter (specific filter cube characteristics not provided).

## 3 Results and Discussion

### 3.1 Characterization of Coupled Carbon Dots

Blue-emissive citric acid-based CDs (CA-CDs) and yellow-emissive urea-doped 1,2-phenylenediamine-based CDs (UPH-CDs) were synthesized by a microwave-assisted method. As illustrated in Figure 1, these individual CDs were subsequently linked using EDC/NHS carbodiimide chemistry to form a structure linked by amide bonds. The resulting coupled material exhibits dual emission characteristics, producing white light under UV irradiation (λ = 366 nm), which indicates the successful combination of the two emissive centers.

**Figure 1:**
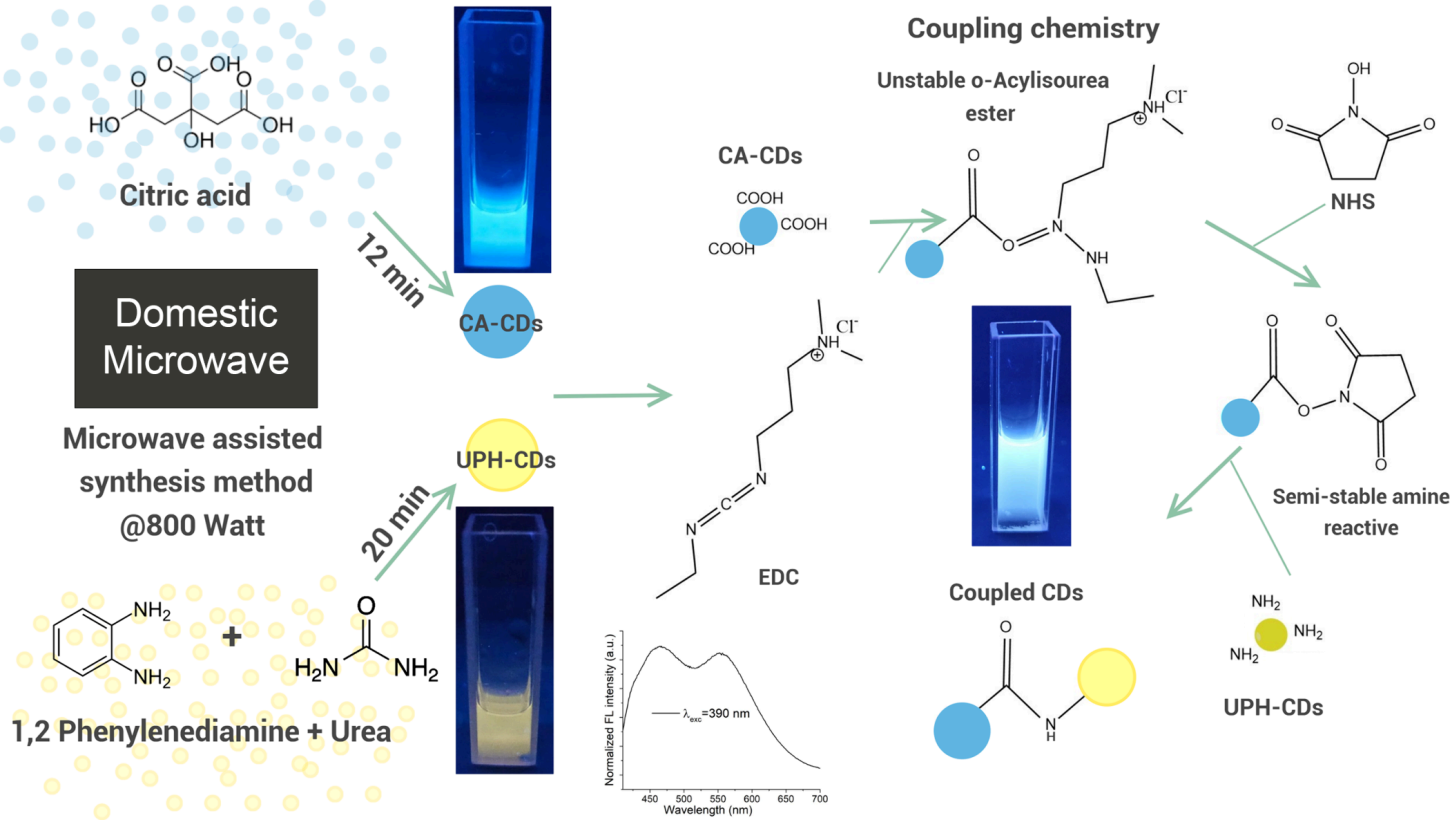
Schematic illustration of the synthesis strategy and sensing principle of the covalently coupled dual-emissive CDs. CA-CDs (blue-emissive, λ_em_ ≈ 455 nm) are synthesized from citric acid, while UPH-CDs (yellow-emissive, λ_em_ ≈ 555 nm) are synthesized separately from *o*-phenylenediamine with urea as an auxiliary nitrogen source. The two preformed carbon dot populations are subsequently covalently linked via carbodiimide-mediated amide bond formation to yield a stable dual-emissive nanostructure and their photoluminescence under UV-light (366 nm) irradiation.

The formation of the covalent linkage was confirmed by FTIR spectroscopy. As shown in Figure 2a, the spectrum of the coupled CDs exhibits characteristic amide peaks at 1638 cm^–1^ (Amide I, C=O stretching) and 1566 cm^–1^ (Amide II, N–H bending). The absence of these peaks in the spectra of the individual CA-CDs and UPH-CDs is highly consistent with the formation of amide linkages between the particles. The CA-CD spectrum displays characteristic carboxyl peaks (C=O at 1695 cm^–1^ and broad −OH stretching around 3400 cm^–1^), while the UPH-CD spectrum features primary and secondary amine N–H stretches in the 3300–3400 cm^–1^ range. Furthermore, the attenuation of the peak at 1703 cm^–1^, corresponding to the asymmetric C=O stretch of free NHS (Figure S1), indicates the consumption of NHS-active esters, further supporting the occurrence of the coupling reaction. Collectively, these spectral features provide strong evidence for the structural model of covalent amide bonds linking the carboxyl-functionalized CA-CDs and the amine-functionalized UPH-CDs.

**Figure 2:**
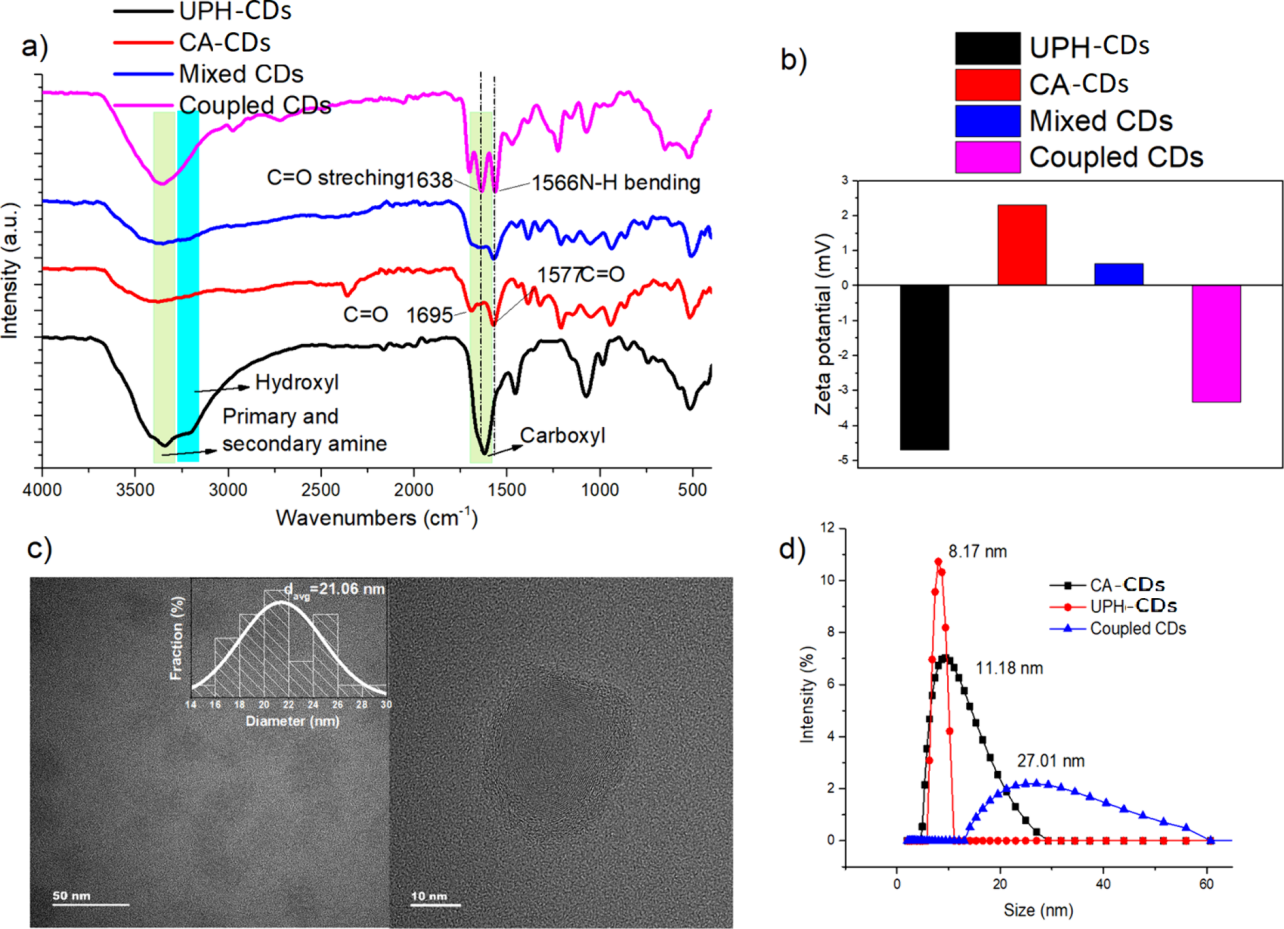
Structural and morphological characterization of the precursor and coupled carbon dots. (a) FTIR spectra of UPH-CDs (black), CA-CDs (red), mixed CDs (blue), and coupled CDs (magenta) showing the vibrational modes of functional groups. (b) Zeta potential values of the four CDs in aqueous dispersion. (c) TEM images of the coupled CDs; the inset displays the statistical size distribution histogram (*d*_avg_ = 21.06 nm). (d) Hydrodynamic size distribution (DLS) comparison of CA-CDs (11.18 nm), UPH-CDs (8.17 nm), and the significantly larger coupled CDs (27.01 nm).

The surface charge properties of the CDs in PBS (pH 7.4) were evaluated using zeta potential measurements (Figure 2b). The CA-CDs exhibited a positive surface charge, whereas the UPH-CDs were negatively charged. While citric acid-based nanodots often exhibit negative charges due to surface carboxyl groups, the slightly positive ζ-potential observed here (+2.3 mV at pH 7.4) is attributed to the specific microwave-induced carbonization degree, which can result in surface states or core-dominated potentials that deviate from typical negatively charged profiles. This positive surface characteristic is a key factor in facilitating the initial proximity for subsequent covalent coupling with the negatively charged UPH-CDs. In the physical mixture, these opposing charges likely induce electrostatic aggregation, resulting in a neutral potential. Notably, the covalently coupled CDs displayed a net negative surface charge comparable to that of UPH-CDs. This behavior suggests a potential core–shell-like arrangement, wherein the positively charged CA-CDs may serve as seeds for the covalent attachment of the negatively charged UPH-CDs. This proposed arrangement, which resembles a core–shell structure, may provide more accessible active sites, thereby facilitating a more efficient interaction with the Cr­(VI) ions. The morphology and size of the coupled CDs were further characterized via TEM. As depicted in Figure 2c, the coupled CDs exhibit a spherical morphology with good dispersion. Statistical analysis of the TEM images, presented in the size distribution histogram (inset of Figure 2c), reveals an average particle diameter of 21.06 nm. These morphological data are in excellent agreement with the DLS results and confirm the successful assembly of the precursor dots into the coupled hybrid system. To further evaluate the size distribution in an aqueous environment, DLS measurements were conducted (Figure 2d). The CA-CDs and UPH-CDs displayed hydrodynamic diameters (*D*_h_) centered at approximately 11.18 and 8.17 nm, respectively. Notably, the covalently coupled CDs exhibited a significantly larger *D*_h_ value of 27.01 nm. This increase corroborates the formation of a larger coupled nanostructure, where the slightly larger size in DLS compared with TEM is attributed to the hydration layer in the aqueous dispersion.

The steady-state optical properties were investigated via UV–vis absorption and photoluminescence spectroscopy (Figure 3a–d). For comparative clarity, both absorbance and emission spectra were normalized to their respective maximum values (*Y*/*Y*_max_) and plotted on a single dimensionless *y* axis. This superimposed presentation is utilized qualitatively to evaluate the spectral overlap between the absorbance of the analyte and the emission of the probe, a critical step in identifying the quenching mechanism (e.g., IFE), while absolute photoluminescence efficiency was discussed separately through quantum yield calculations. The UPH-CDs displayed a distinct absorbance peak at 420 nm, whereas the coupled CDs exhibited a peak at 440 nm. The mixed CDs showed a slightly broader peak centered at 450 nm, while the CA-CDs exhibited absorbance in this region without a defined peak. Subsequently, the photoluminescence (PL) properties were investigated under varying excitation wavelengths. Upon excitation at 380 nm, both the CA-CDs and the mixed CDs exhibited emission maxima at 455 nm (Figure 3b,c). UPH-CDs maintained a stable emission peak position around 565 nm under both 380 and 410 nm excitation. This positional stability is essential for the coupled system, as it ensures a consistent spectral signature for the yellow-emissive component across the usable excitation range (Figure 3d). Crucially, the coupled CDs excited at 380 nm revealed two distinct emission peaks at approximately 458 and 554 nm (Figure 3a, red line), confirming the successful formation of a dual-emissive system. When the excitation wavelength was changed to 410 nm, the emission peak of CA-CDs and mixed CDs red-shifted to approximately 470 nm (Figure 3b,c). UPH-CDs maintained a similar emission peak around 565 nm under 380 and 410 nm excitation independently (Figure 3d). For the coupled CDs excited at 410 nm (Figure 3a, blue line), the primary emission peak was observed at ∼475 nm, with the secondary peak at 555 nm appearing as a significantly less intense broad shoulder. These peaks correspond to the characteristic emissions of the CA-CD and UPH-CD components, respectively. By contrast, the simply mixed CDs exhibited only a single emission peak dominated by the CA-CD component (Figure 3b), highlighting the structural advantage of the coupled system.

**Figure 3:**
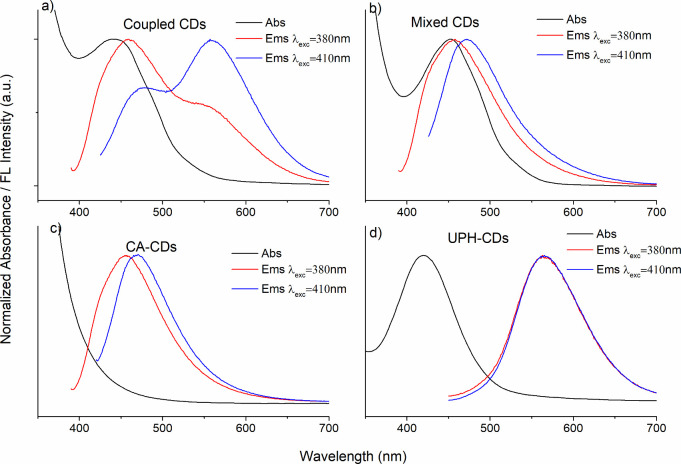
Steady-state optical properties of the carbon dot samples. (a–d) Normalized UV–vis absorption (black lines) and normalized photoluminescence (PL) emission spectra (red and blue lines) of UPH-CDs, CA-CDs, mixed CDs, and coupled CDs. Both absorbance and intensity values are normalized to their respective maximum values (*Y*/*Y*_max_) for qualitative spectral overlap analysis. The excitation wavelengths used for PL are λ_exc_ = 380 nm for the red line PL emission peak and λ_exc_ = 410 nm for the blue line PL emission peak.

The quantum yields of CA-CDs, UPH-CDs, and coupled CDs were calculated to be 7.37%, 1.29%, and 4.2%, respectively, in reference to Coumarin 102 as the standard (QY = 0.93). The obtained quantum yield values are comparatively low, which can be attributed to the presence of abundant surface states and nonradiative decay pathways typically observed in carbon-dot-based systems.^40–42^ Nevertheless, the Cr­(VI)-induced quenching is highly efficient, indicating that sensing performance in this case is governed mainly by the interaction between Cr­(VI) and the emissive centers rather than by the absolute photoluminescence efficiency.

The fluorescence spectrum in Figure 4b further illustrates that the coupled CDs exhibited dual photoluminescence emission peaks when excited with wavelengths between 370 and 420 nm. Notably, the emission peak originating from UPH-CDs (the right-side peak) was not apparent in the mixed CDs prepared by simple mixing under identical conditions. When the coupled CDs were excited at 390 nm, distinct dual emission peaks were observed, with the CA-CDs’ emission peak showing a 10 nm red-shift and the UPH-CDs’ emission peak exhibiting a 10 nm blue-shift compared to their individual states. The most prominent dual emissions for coupled CDs were observed with excitation at 380 nm and, as suggested by Figure 4b, around 410 nm. In contrast, for mixed CDs, secondary interactions like electrostatic forces likely persisted, leading to quenching of the UPH-CD emission (Figure 4a). The covalent linkage formed via EDC/NHS chemistry is hypothesized to act as a spacer, potentially maintaining a beneficial spatial distribution between the two CD populations. This engineered architecture prevents the close-contact aggregation seen in the mixture, thereby preserving the intrinsic emissive properties of both nanoparticle types and enabling stable dual fluorescence. This result underscores the superiority of the covalent coupling approach for designing stable, multiemissive nanomaterials. The PL spectra of the coupled and mixed CDs excited at more frequent intervals are shown in Figure S2.

**Figure 4:**
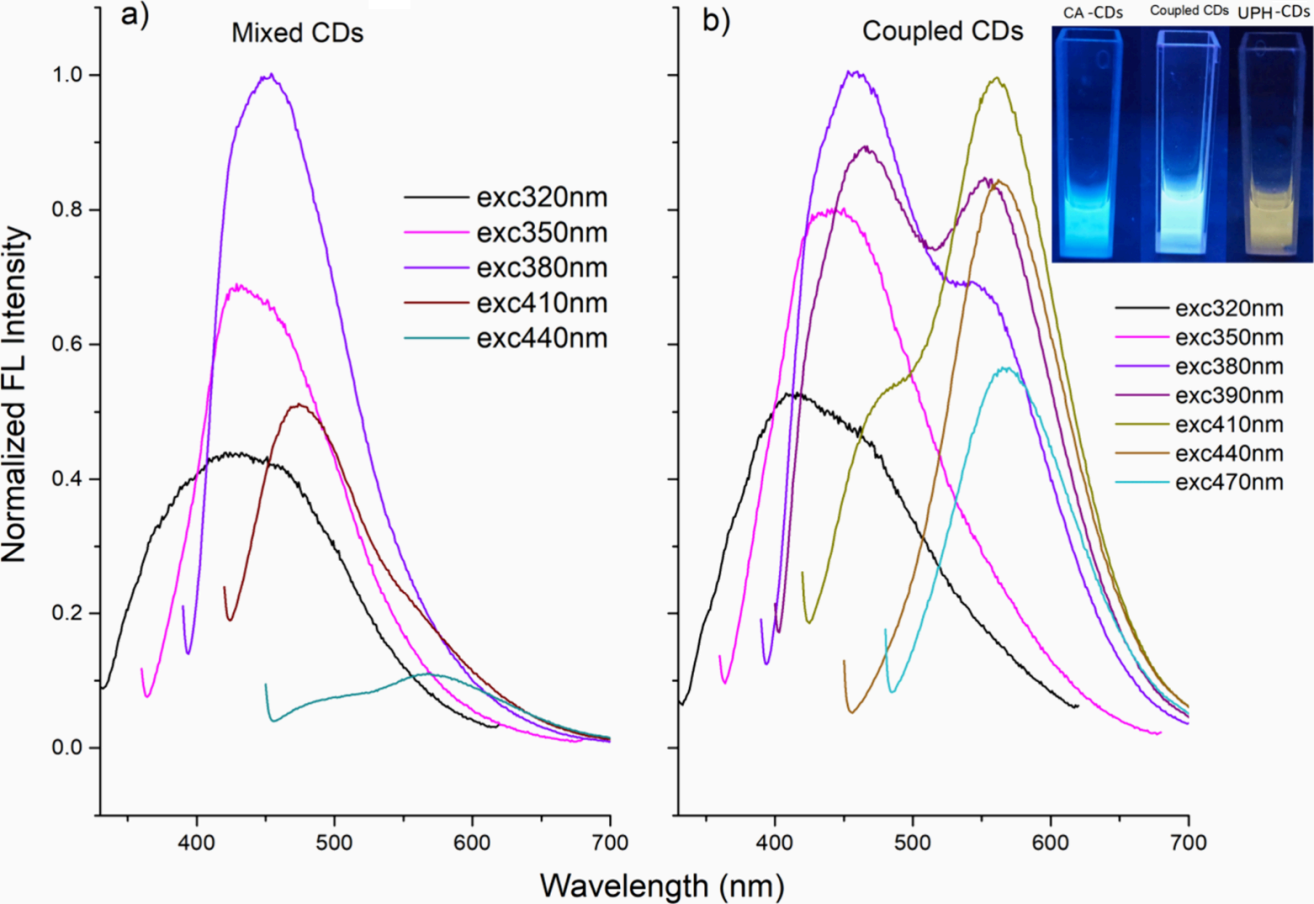
PL spectra of (a) mixed CDs with different excitation wavelengths (320–440 nm) and (b) coupled CDs with different excitation wavelengths (320–470 nm). The inset figure shows the photoluminescence of CA-CDs (left), coupled CDs (middle), and UPH-CDs (right) under UV-light (366 nm) irradiation.

The photostability of the CDs was evaluated under continuous illumination (λ_exc_ = 380 nm) for 1 h (Figure 5a) and monitored over a period of 20 days under both 380 and 410 nm excitation (Figure 5b). All CD variants exhibited excellent stability during the 1 h continuous illumination test (λ_exc_ = 380 nm). In the long-term stability assessment over 20 days, the coupled CDs excited at 380 nm demonstrated the highest retention of fluorescence intensity. Conversely, the UPH-CDs exhibited a more pronounced decline in stability over the 20 day period when excited at 410 nm. This instability of the UPH-CD component likely accounts for the reduced stability observed in the coupled CDs under 410 nm excitation compared to 380 nm. Meanwhile, both the CA-CDs and the mixed CDs, which exhibit their emission maxima under 380 nm excitation, showed negligible fluorescence loss over the 20 days. Furthermore, the mixed CDs maintained high stability under 410 nm excitation.

**Figure 5:**
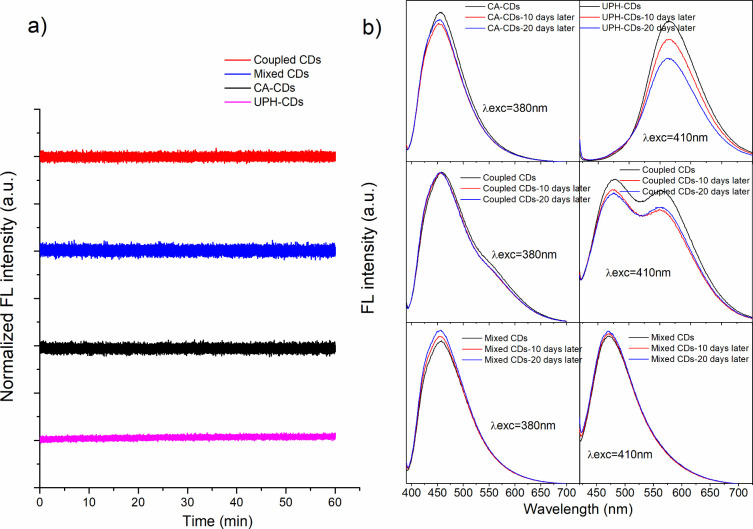
(a) Photostability of CDs for 1 h upon continuous illumination with λ_exc_ = 380 nm and (b) stability of CDs for 20 days with λ_exc_ = 380 and 410 nm.

### 3.2 Cr­(VI) Sensing

The fluorescence quenching behavior of the coupled CDs in the presence of varying Cr­(VI) concentrations was investigated to evaluate their sensing potential. All sensing experiments were performed in triplicate (*n* = 3) to ensure statistical significance. A clear linear relationship was observed between the relative fluorescence intensity (*I*_0_/*I*) at 455 nm and the Cr­(VI) concentration, where *I*_0_ and *I* represent the initial fluorescence intensities in the absence and presence of the quencher, respectively. The error bars in the calibration plot represent the standard deviation calculated from these independent measurements. This linear correlation held within the Cr­(VI) concentration range of 5 to 500 μM (Figure 6b). The limit of detection (LOD) for Cr­(VI) was determined to be 4.6 μM, based on three times the standard deviation of the blank solution (*R* ^2^ = 0.99). The LOD (4.6 μM) is situated in close proximity to the lower limit of the established linear range (5 μM). This convergence indicates that the probe exhibits high sensitivity and statistical reliability. When excited at 410 nm, Cr­(VI) sensing was also observed, with a slightly higher LOD of 5.9 μM. For comparison, mixed CDs and individual CA-CDs showed quenching with a minimum of 15.6 μM Cr­(VI) ions. To compare the interaction of UPH-CDs and CA-CDs with Cr­(VI) ions, the effect of quenching their maximum emissions at the same concentration was examined. 125 μM Cr­(VI) quenched CA-CDs by 21%, while UPH-CDs quenched by 15% in an approximately less than 15 s response time (Figure S3). Although the quantum yield of the coupled CDs (4.2%) is lower than that of the precursor CA-CDs (7.37%), the analytical sensing performance toward Cr­(VI) is significantly improved. The superior sensitivity is evidenced by the nearly 3.4-fold enhancement in the limit of detection (4.6 μM vs 15.6 μM). This indicates that the performance of the sensor is governed by the effectiveness of the quenching interaction and the structural accessibility of the emissive sites within the coupled architecture, rather than the absolute photoluminescence efficiency. Thus, the covalent coupling represents a successful structural innovation aimed at optimizing sensing functionality over mere luminous brightness.

**Figure 6:**
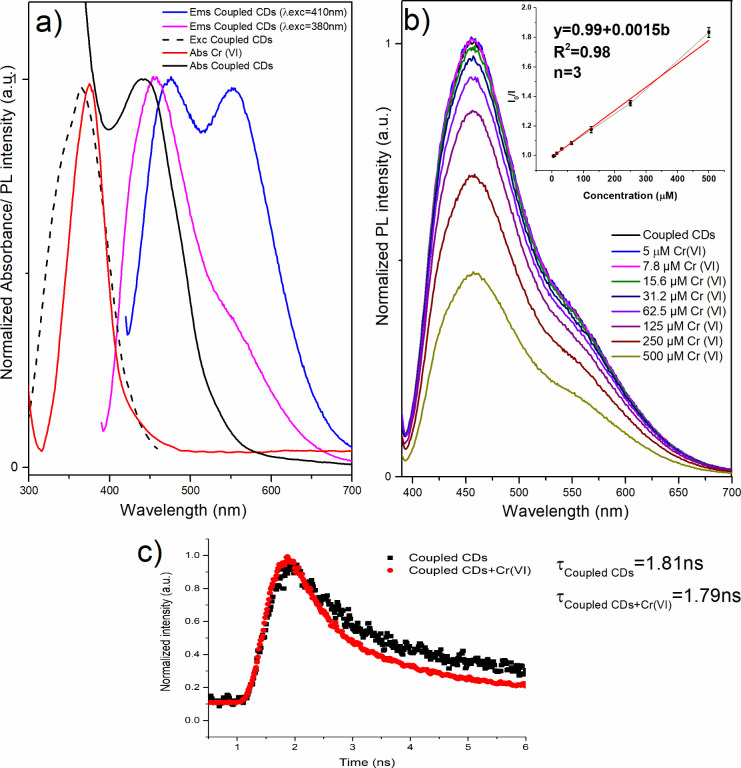
Fluorescence quenching performance of the coupled CDs for Cr­(VI) detection. (a) Spectral overlap between the absorbance spectrum of Cr­(VI) and the emission of the coupled CDs, indicating the inner filter effect (IFE). (b) Fluorescence response of the probe to increasing concentrations of Cr­(VI) (5–500 μM); the inset shows the corresponding linear calibration plot. Error bars represent the standard deviation of three independent measurements (*n* = 3). (c) Time-resolved fluorescence decay curves in the absence and presence of Cr­(VI), showing an invariant average lifetime (τ_Coupled CDs_ = 1.81 ns and τ_Coupled CDs+Cr(VI)_ = 1.79 ns).

In principle, the dual-emissive nature of the coupled CDs allows for the use of ratiometric sensing. However, as demonstrated in Figure S3, the UPH-CD-related emission at 555 nm also undergoes approximately 15% quenching upon the addition of Cr­(VI). This means that the 555 nm band cannot act as a true internal reference. Since both emission bands respond to Cr­(VI), the calculated *I*_455_/*I*_555_ ratio does not improve sensitivity or linearity compared with single-band analysis. It is important to note that while the dual-emissive nature of the coupled CDs provides a unique optical signature (e.g., white light emission), the significant spectral overlap of Cr­(VI) absorbance with both emission peaks leads to a dual-signal quenching effect. Consequently, the 555 nm band does not function as a nonresponsive internal reference, and the sensing performance is more accurately represented by the single-band quenching of the highly responsive 455 nm emission. For this reason, the sensing performance was primarily evaluated using the more responsive 455 nm emission band (Figure 6b). Furthermore, the dual-emissive nature that yields white light emission provides a significant advantage for qualitative visual sensing. Unlike single-emissive probes, where Cr­(VI) only induces a decrease in brightness, the differential quenching of the coupled CD bands results in a detectable change in the overall emission color. This ‘color-tuning’ response allows the developed probe to function as a visual indicator for Cr­(VI) detection in environmental monitoring, where a shift from the initial white light can be observed even without sophisticated spectrofluorometric equipment.

Cr­(VI) ions (250 μM) gave a damping response as soon as it was added to the coupled CDs. This shows that the response time of the coupled CDs is quite short (<15 s) (Figure 7a). The selectivity of coupled CDs toward Cr­(VI) was investigated, including metal ions of Cr^3+^, Mn^2+^, Co^2+^, Cu^2+^, Fe^2+^, Fe^3+^, Hg^2+^, Cd^2+^, Ni^2+^, Ag^+^, and Zn^2+^. To evaluate the selectivity of coupled CDs to other metal ions (500 μM), these ions were added into coupled CDs, and their corresponding fluorescence emission spectra were recorded at 380 nm excitation. Cr­(VI) ions induced a significant quenching of the coupled CDs’ fluorescence, reducing the intensity to approximately 58% of the initial value (representing a quenching efficiency of about 42%) at 455 nm emission band. In stark contrast, most other metal ions caused minimal changes to the fluorescence intensity. For instance, Zn­(II) ions resulted in a fluorescence intensity of approximately 103% (relative to coupled CDs alone at 100%), indicating negligible quenching or even a slight enhancement effect. This demonstrates a pronounced selectivity of the coupled CDs for Cr­(VI) over Zn­(II) and the other tested metal ions. Notably, the probe could also effectively distinguish toxic Cr­(VI) from the much less toxic Cr­(III), which caused significantly less quenching.

**Figure 7:**
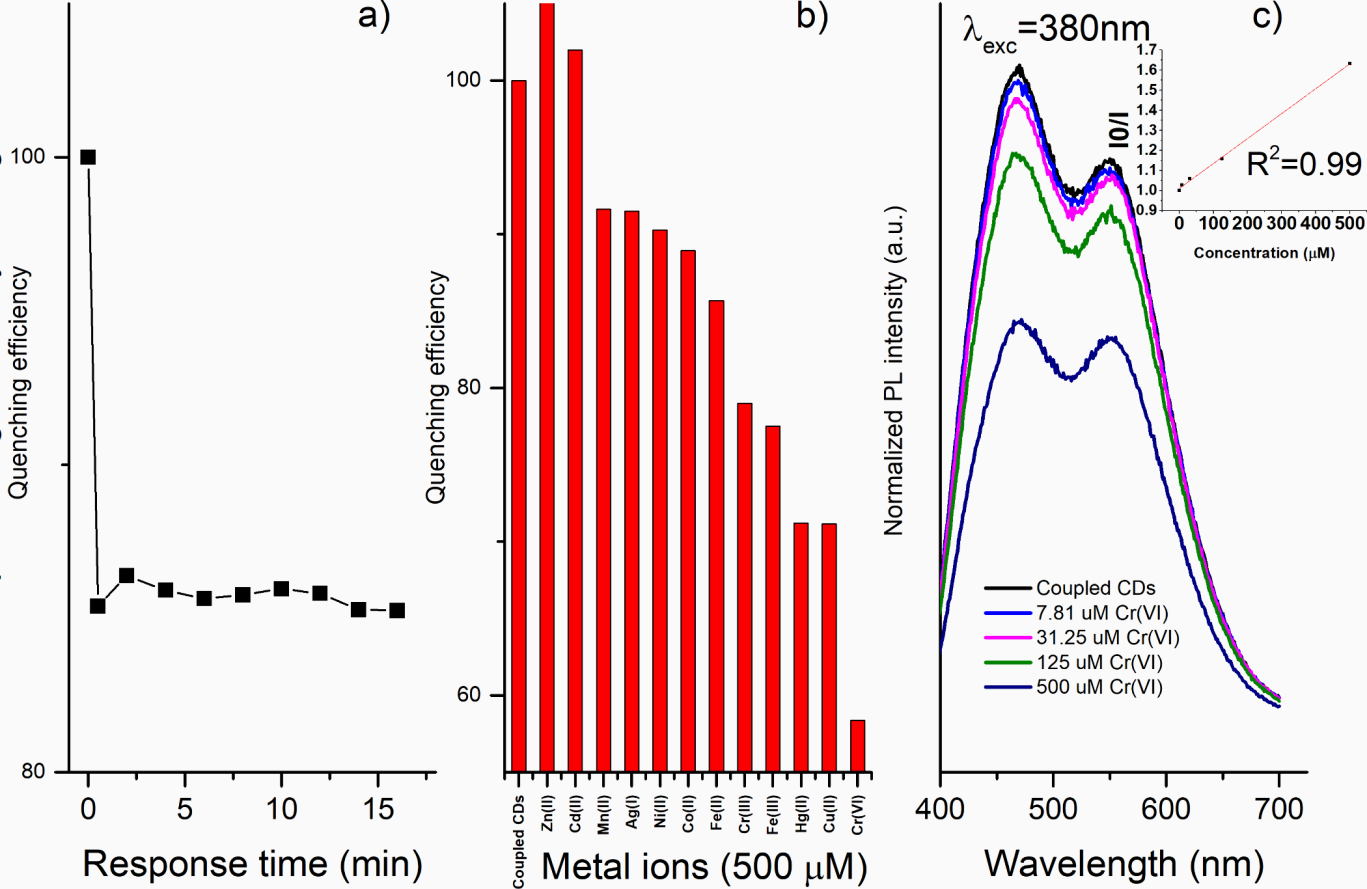
(a) Response time to 250 μM Cr­(VI) sensing and (b) selectivity to other metal ions with 500 μM. (c) Linear relationship between fluorescence intensity and Cr­(VI) concentration in tap water samples.

The observed fluorescence quenching of the coupled CDs by Cr­(VI), especially the pronounced decrease in the 455 nm emission from the CA-CD component, prompts an examination of the sensing mechanism. As illustrated in Figure 6a, Cr­(VI) displays strong absorbance that encompasses both the excitation window (λ_exc_ = 380 nm) and the primary emission peak of the coupled CDs (λ_ems_ = 455 nm). This spectral overlap strongly suggests that an inner filter effect (IFE) is a key contributor to the fluorescence quenching. The IFE observed in this system can be categorized into primary and secondary processes. The primary IFE occurs as Cr­(VI) ions that compete with the carbon dots to absorb the excitation photons at 380 nm. Simultaneously, a secondary IFE takes place because the emission band of the coupled CDs (455 nm) significantly overlaps with the broad absorbance spectrum of Cr­(VI) (Figure 6a), leading to the reabsorption of emitted fluorescence by the analyte. This dual-mode optical interference results in the rapid and pronounced decrease in the measured fluorescence intensity.

However, IFE alone may not account for the total quenching observed. To distinguish between static and dynamic quenching pathways, we performed fluorescence lifetime measurements of the coupled CDs in the absence and presence of Cr­(VI) (Figure 6c). The average fluorescence lifetime (τ_avg_) of the coupled CDs was calculated to be 1.81 ns. Crucially, upon the addition of a quenching concentration of Cr­(VI) (250 μM), the fluorescence lifetime remained virtually unchanged (1.79 ns). This mechanism is well-documented for Cr­(VI) detection using various carbon-based nanomaterials. The differential quenching behavior, where the CA-CD emission at 455 nm is more substantially quenched than the UPH-CD emission at 555 nm, is crucial. This is likely due to a greater degree of spectral overlap between the 455 nm emission and the Cr­(VI) absorbance spectrum compared to the 555 nm emission band.

To investigate the quenching mechanism, we integrated our time-resolved fluorescence data with recent comparative studies in the literature. As reported in the comprehensive study by Luo et al. using *Poria cocos*-derived CDs, the quenching mechanisms for metal ions can be distinct; they demonstrated that Cr­(VI) sensing is primarily governed by the IFE due to strong spectral absorption, whereas Fe­(III) sensing relies on static quenching complexation.^45^ This distinction supports our observation of the significant spectral overlap between Cr­(VI) absorption and the CD emission (Figure 6a). Furthermore, Wu et al. recently established for Fe­(III)-CD systems that static quenching is characterized by temperature-independent quenching constants and, crucially, invariant fluorescence lifetimes.^46^ In our study, the fluorescence lifetime of the coupled CDs remained virtually unchanged (τ_Coupled CDs_ = 1.81 ns and τ_Coupled CDs+Cr(VI)_ = 1.79 ns) in the presence of Cr­(VI) (Figure 6c). Aligning our lifetime data with the kinetic models validated by Wu et al. and the mechanistic classification by Luo et al., we conclude that the signal response toward Cr­(VI) is not due to dynamic quenching but is primarily governed by a dominant inner filter effect. While the invariant lifetime measurements (Figure 6c) clearly distinguish this from dynamic processes, the quenching is predominantly an optical phenomenon arising from the spectral absorption by Cr­(VI) ions. A significant advantage of this coupled CD probe is its rapid response time, with fluorescence quenching occurring in less than 15 s upon addition of Cr­(VI) (Figure 7a). This is considerably faster than many other CD-based Cr­(VI) sensors recently reported in the literature (Table 1). Several factors likely contribute to this fast response. First, IFE, as a proposed dominant mechanism, is an optical phenomenon and is inherently rapid, not being limited by slow chemical reaction kinetics. Second, the excellent water solubility and good dispersibility of the covalently coupled CDs likely facilitate the rapid diffusion of Cr­(VI) ions to the CD surface, enabling quick and efficient interaction. The covalent linkage itself might also contribute to a stable and accessible structure for Cr­(VI) interaction, particularly with the CA-CD components. The immediate quenching observed indicates an efficient process without slow intermediate steps, underscoring the potential of these coupled CDs for real-time Cr­(VI) detection applications. To investigate the preliminary feasibility of the coupled CD probe in practical settings, tap water was utilized as a test matrix. Known concentrations of Cr­(VI) were spiked into the tap water samples. The observed linear relationship (*R* ^2^ = 0.99) in the tap water matrix (Figure 7c) indicates that the sensing mechanism is maintained in the presence of common domestic water ions. While these results show promising potential for Cr­(VI) monitoring in laboratory tap water, further comprehensive validation across diverse environmental matrices remains to be explored in future studies. Furthermore, our studies on pH effects demonstrate that while Cr­(VI) quenching is pH-dependent, the probe remains highly effective under the near-neutral conditions (pH = 7.0–7.4) typical of biological and drinking water environments.

**Table 1:** Comparison of Other Fluorescence-Based Sensors Using CDs for Cr­(VI) Detection^a^

fluorescent sensing probe carbon dots	production method of QDs	response mechanism/response time to Cr(VI) ions	detection range (μM)	LOD (μM)	**year** ^ref^
orange peel-derived CDs modified with EDTA	one-step green hydrothermal	quenching-IFE/60 min	0.01–50	0.01	2020 ^43^
N-rich CDs (Citric acid and urea-based)	microwave-assisted	quenching-IFE/NA	10–100	3.2	2021 ^7^
B–N codoped CDs (APBA based)	one-step hydrothermal	quenching- IFE/NA	0–100	0.41	2021 ^10^
chitosan-based CDs	one-step hydrothermal	quenching- IFE/20 min	10–300	2.83	2022 ^44^
succinic acid and tris-based CDs	one-step hydrothermal	quenching/IFE 5 min	1–125	0.6	2024 ^35^
green tea leaf-mediated CDs	one-step hydrothermal	quenching- IFE/5 min	0.2–150	0.07	2024 ^34^
S–N codoped CDs (Citric acid and thiourea-based)	one-step hydrothermal	quenching- IFE/5 min	0–100	0.686	2024 ^33^
CDs derived from *Lycium barbarum*	one-step hydrothermal	quenching- IFE/1 min	0–40	0.16	2024 ^31^
CDs derived from *Poria cocos*	one-step hydrothermal	quenching- IFE/NA	0–300	1.07 × 10^–3^	2025 ^45^
dual-emissive coupled CDs	microwave-assisted	quenching/less than 15 s	5–500	4.6	this work

a APBA: 3-Aminophenylboronic acid, EDTA: ethylenediaminetetraacetic acid, IFE: inner filter effect, SiQDs@PAMAM–OH: silicon quantum dots, coated by hydroxyl poly­(amidoamine) dendrimer, Tris: tris­(hydroxymethyl)­aminomethane, NA: not available.

The effect of pH on the Cr­(VI)-induced fluorescence quenching was investigated to assess the sensing behavior of the coupled CDs under different environmental conditions (Figure S4). In these experiments, potassium chromate (K_2_CrO_4_) was used as the Cr­(VI) source, for which chromate ions (CrO_4_^2–^) are the dominant species under neutral and basic conditions. As the pH increases, the quenching efficiency gradually increases, which can be attributed to the enhanced stability and optical absorption of CrO_4_^2–^, thereby strengthening the inner filter effect. In addition, partial deprotonation of surface functional groups on the CDs at higher pH values may further facilitate the interaction between the emissive centers and the Cr­(VI). Despite the stronger quenching observed under more basic conditions, sensing experiments involving real samples were intentionally conducted at approximately pH 7, and cell imaging experiments were performed at pH 7.4. These conditions were selected to ensure environmental relevance and physiological compatibility, demonstrating that the probe operates effectively under realistic rather than highly optimized pH conditions.

A significant advantage of the developed coupled CD probe is its exceptionally rapid response with fluorescence quenching occurring in less than 15 s upon the addition of Cr­(VI). This efficiency can be attributed to several synergistic factors. First, as the dominant mechanism, the IFE is an optical phenomenon that is inherently near-instantaneous and is not limited by slow chemical reaction kinetics or molecular rearrangements. Second, the excellent water solubility and dispersibility of the covalently coupled CDs facilitate the rapid diffusion of Cr­(VI) ions to the particle surface. Finally, the stable, covalently linked architecture may provide highly accessible active sites, particularly on the CA-CD components, enabling an immediate interaction with the analyte. This rapid detection capability underscores the potential of the probe for near-real-time environmental monitoring applications.

### 3.3 Cr­(VI) Detection in the NIH/3T3 Cell Line

The probe’s ability to detect Cr­(VI) ions in cellular environments was evaluated through cytotoxicity testing and fluorescence microscopy. Before cellular introduction of the probe, we first conducted cytotoxicity assays to establish the concentration that could be safely used. Figure 8e presents the cytotoxicity results for NIH/3T3 cells after a 4 h exposure to varying concentrations of coupled CDs. Cell viability remained consistent and high (above 90%) across the tested concentrations from 0.1 to 5 mg/mL, indicating no significant acute cytotoxicity within the 4 h test window.

**Figure 8:**
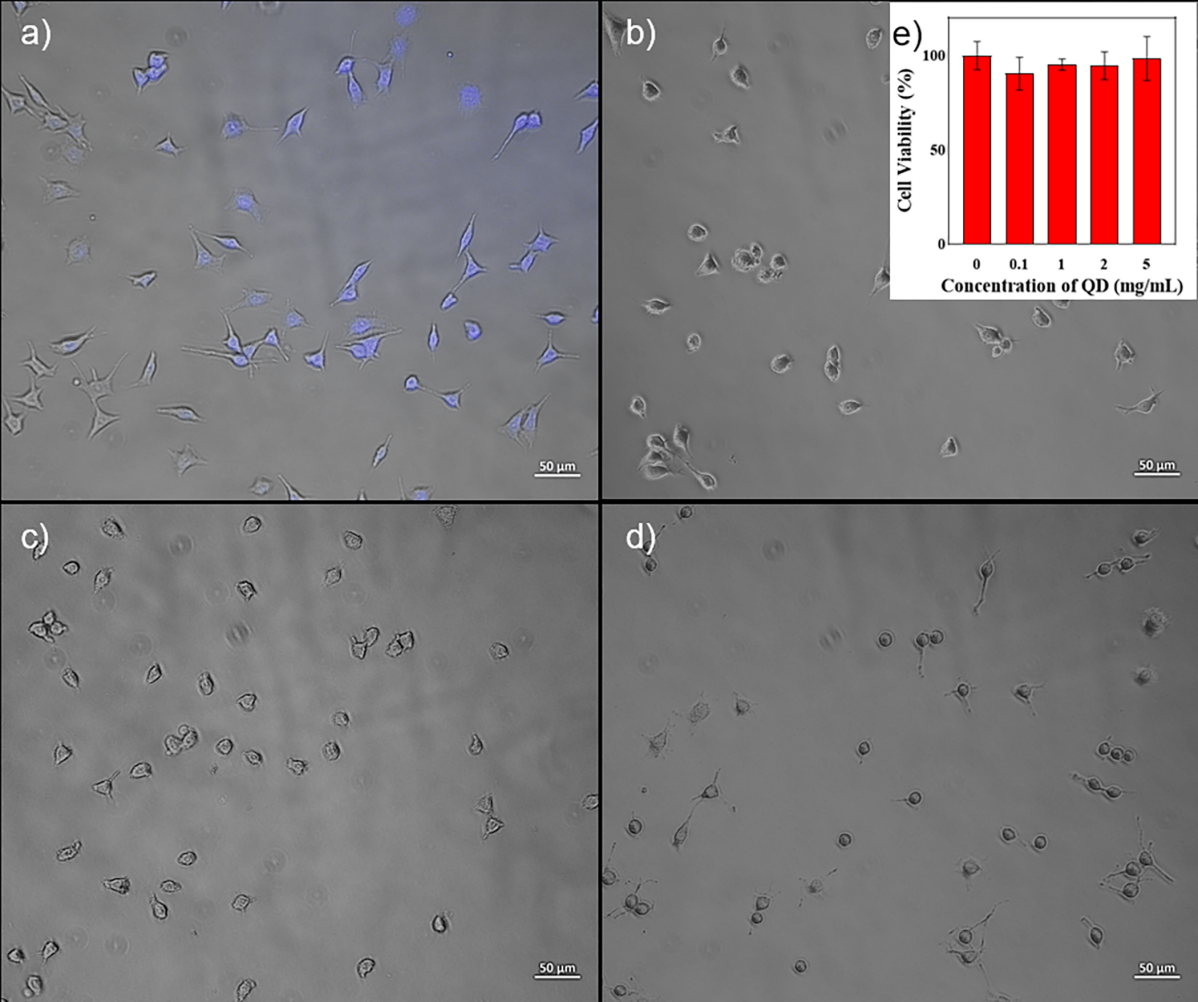
Merged images of 3T3 cells: (a) cells treated with the probe for 1 h, cells treated with the probe for 1 h followed by Cr­(VI) at concentrations of (b) 50 μM and (c) 250 μM for an additional 2 h, and (d) control cells with no probe and no Cr­(VI) (scale bar, 50 μm). (e) The effect of the probe at various concentrations (0–5 mg/mL) on the viability of the 3T3 cell line. All data were recorded as the means ± SD for *n* = 3.

The sensing capability of the synthesized probe (at a concentration of 2 mg/mL) was evaluated using NIH/3T3 cells. Figure 8 displays merged bright-field and fluorescence microscopy images representative of these experiments. Control groups, lacking either the probe or Cr­(VI), showed no fluorescence. Cells incubated solely with the probe exhibited fluorescence. However, when cells pretreated with the probe were subsequently exposed to Cr­(VI) solution, the fluorescence intensity gradually weakened over time and was completely quenched after 2 h. In conclusion, the probe demonstrates low acute cytotoxicity to NIH/3T3 cells under the experimental conditions used for imaging and offers effective Cr­(VI) detection potential through fluorescence quenching.

## 4 Conclusions

In summary, this study demonstrates the structural assembly of a dual-emissive CD system via covalent coupling of CA-CDs and UPH-CDs. While the probe exhibits a distinct fluorescence quenching response toward Cr­(VI) with a limit of detection of 4.6 μM, the dual emission architecture functions primarily as a qualitative visual transducer rather than a quantitative ratiometric reference. Although both peaks respond to the presence of Cr­(VI), the high sensitivity of the 455 nm band allows for a robust and rapid detection system. These novel hybrid CDs functioned as a selective fluorescent probe for Cr­(VI) ions, distinguishing them from a range of other metal ions. The sensing system demonstrated a linear detection range for Cr­(VI) from 5 to 500 μM, with a calculated limit of detection of 4.6 μM. A significant advantage of this probe is its rapid response time, detecting Cr­(VI) in under 15 s. The covalent coupling was crucial for achieving a stable dual-emissive architecture and enhanced analytical sensing performance (e.g., lower LOD and rapid response) compared to standalone or simply mixed CDs. While the structural coupling results in a moderate decrease in absolute quantum yield, the synergistic effect of the dual-signal system significantly improves the reliability and sensitivity of Cr­(VI) detection. Furthermore, the developed CDs were successfully applied for Cr­(VI) detection within NIH/3T3 cells using fluorescence imaging. Importantly, these probes showed high cell viability (>90%) for the NIH/3T3 cell line during short-term (4 h) exposure at concentrations up to 5 mg/mL, supporting their suitability for rapid *in vitro* bioimaging applications. The covalent coupling was crucial for the stable dual-emissive properties and enhanced performance compared to simply mixed CDs. These results highlight the potential of these coupled CDs as a platform for developing Cr­(VI) detection systems. Although this work focuses on the structural assembly and fundamental sensing properties, the stability of the probe in tap water and cell media highlights its versatility for further analytical optimization.

## Supplementary Material



## Notes

FTIR spectrum of pure NHS (Figure S1); photoluminescence (PL) spectra of the coupled and mixed CDs excited at frequent intervals (Figure S2); quenching effect of Cr­(VI) on the maximum emissions of UPH-CDs and CA-CDs and their response time (Figure S3); effect of pH on the Cr­(VI)-induced fluorescence quenching of the coupled CDs (Figure S4) (PDF)

## References

[1] Li W., Han Y., Wang X., Zhao H. (2024). Double Emissions in Boron-Doped Carbon
Dots for Anticounterfeiting Applications. ACS
Appl. Nano Mater..

[2] Zhang L., Wang Y., Jia L., Zhang X., Xu J. (2023). Dynamic Anti-Counterfeiting
and Reversible Multi-Level Encryption-Decryption Based on Spirulina
Derived PH-Responsive Dual-Emissive Carbon Dots. J. Lumin..

[3] Kumari R., Kumar A., Negi K., Sahu S. K. (2023). Multicolor-Emissive
Carbon Dots for White-Light-Emitting Diodes and Room-Temperature Phosphorescence. ACS Appl. Nano Mater..

[4] Chen L., Zheng J., Du Q., Yang Y., Liu X., Xu B. (2020). Orange-Emissive Carbon
Dot Phosphors for Warm White Light-Emitting
Diodes with High Color Rendering Index. Opt.
Mater. (Amst)..

[5] Guan Y., Lu Y., Wei Y. (2024). Fabrication of a Ratiometric
Fluorescent Probe Based
on Tb3+ Doped Dual-Emitting Carbon Dots for the Detection of Cytochrome
c. Spectrochim. Acta A Mol. Biomol. Spectrosc..

[6] Wang C., Huang Y., Jiang K., Humphrey M. G., Zhang C. (2016). Dual-Emitting
Quantum Dot/Carbon Nanodot-Based Nanoprobe for Selective and Sensitive
Detection of Fe3+ in Cells. Analyst.

[7] Bogireddy N.
K. R., Sotelo
Rios S. E., Agarwal V. (2021). Simple One Step Synthesis
of Dual-Emissive Heteroatom Doped Carbon Dots for Acetone Sensing
in Commercial Products and Cr (VI) Reduction. Chemical Engineering Journal.

[8] Chu H., Yao D., Chen J., Yu M., Su L. (2021). Detection
of Hg2+by
a Dual-Fluorescence Ratio Probe Constructed with Rare-Earth-Element-Doped
Cadmium Telluride Quantum Dots and Fluorescent Carbon Dots. ACS Omega.

[9] Ma Y., Chen Y., Liu J., Han Y., Ma S., Chen X. (2018). Ratiometric Fluorescent Detection
of Chromium­(VI) in Real Samples
Based on Dual Emissive Carbon Dots. Talanta.

[10] Jia M., Peng L., Yang M., Wei H., Zhang M., Wang Y. (2021). Carbon Dots with Dual Emission: A
Versatile Sensing Platform for
Rapid Assay of Cr (VI). Carbon N. Y..

[11] Li Y. f., Zhang X., Lu Q., Cao J. z., Gao S., Liu Q. z., Cai X. x., Zhao H. (2024). Cellulose-Based Yellow-Green
Emitting Carbon Dots with Large Stokes Shift as Effective “Turn
off-on” Fluorescence Platforms for Cr (VI) and AA Dual Efficacy
Detection. Anal. Chim. Acta.

[12] Liu J., Li D., Zhang K., Yang M., Sun H., Yang B. (2018). One-Step Hydrothermal
Synthesis of Nitrogen-Doped Conjugated Carbonized Polymer Dots with
31% Efficient Red Emission for In Vivo Imaging. Small.

[13] Pan Y., Xu M., Cai L., Qiao L., Wang S., Zhang K., Ran X., Guo L. (2024). Red Dual-Emissive Carbon Dots for Cu2+ Selective Detection
and Dynamical Monitoring. ACS Appl. Nano Mater..

[14] Kumari S., Nehra M., Jain S., Kumar A., Dilbaghi N., Marrazza G., Chaudhary G. R., Kumar S. (2024). Carbon Dots for Pathogen
Detection and Imaging: Recent Breakthroughs and Future Trends. Microchimica Acta.

[15] Ji C., Zhou Y., Leblanc R. M., Peng Z. (2020). Recent Developments
of Carbon Dots in Biosensing: A Review. ACS
Sens..

[16] Yuan C., Qin X., Xu Y., Shi R., Cheng S., Wang Y. (2021). Dual-Signal
Uric Acid Sensing Based on Carbon Quantum Dots and o-Phenylenediamine. Spectrochim. Acta A Mol. Biomol. Spectrosc..

[17] Vallan L., Imahori H. (2022). Citric Acid-Based Carbon
Dots and Their Application
in Energy Conversion. ACS Appl. Electron. Mater..

[18] Arkin K., Zheng Y., Hao J., Zhang S., Shang Q. (2021). Polychromatic
Carbon Dots Prepared from M-Phenylenediamine and Urea as Multifunctional
Fluorescent Probes. ACS Appl. Nano Mater..

[19] Heng C., He B., Wang L. (2024). A Dual-Mode Ratiometric Fluorometric and Colorimetric
Platform Based on Nitrogen-Doped Carbon Dots and o-Phenylenediamine
for the Detection of Nitrite. J. Fluoresc..

[20] Marin R., Ahuja Y., Bose R. N. (2010). Potentially Deadly Carcinogenic Chromium
Redox Cycle Involving Peroxochromium­(IV) and Glutathione. J. Am. Chem. Soc..

[21] Nickens K. P., Patierno S. R., Ceryak S. (2010). Chromium Genotoxicity:
A Double-Edged
Sword. Chem. Biol. Interact..

[22] Cancer, I. A. for R. Chromium, Nickel and Welding.IARC Monogr Eval Carcinog Risks Hum; International Agency for Research on Cancer 1990, 677.

[23] Sperling M., Xu S., Welz B. (2009). Determination of Chromium­(III)
and Chromium­(VI) in
Water Using Flow Injection on-Line Preconcentration with Selective
Adsorption on Activated Alumina and Flame Atomic Absorption Spectrometric
Detection. Anal. Chem..

[24] Liang P., Shi T., Lu H., Jiang Z., Hu B. (2003). Speciation of Cr­(III)
and Cr­(VI) by Nanometer Titanium Dioxide Micro-Column and Inductively
Coupled Plasma Atomic Emission Spectrometry. Spectrochim. Acta Part B At. Spectrosc..

[25] Hagendorfer H., Goessler W. (2008). Separation of Chromium­(III)
and Chromium­(VI) by Ion
Chromatography and an Inductively Coupled Plasma Mass Spectrometer
as Element-Selective Detector. Talanta.

[26] Wang Z., Wang X., Niu W., Feng L. (2017). A Specific Fluorescence
Probe for Chromium (VI) Anions and Its Application. Sens. Actuators B Chem..

[27] Hu X., Chai J., Liu Y., Liu B., Yang B. (2016). Probing Chromium­(III)
from Chromium­(VI) in Cells by a Fluorescent Sensor. Spectrochim. Acta A Mol. Biomol. Spectrosc..

[28] Ye J. H., Wang Y., Bai Y., Zhang W., He W. (2014). A Highly Selective
Turn-on Fluorescent Chemodosimeter for Cr­(vi) and Its Application
in Living Cell Imaging. RSC Adv..

[29] Hosseini M., Gupta V. K., Ganjali M. R., Rafiei-Sarmazdeh Z., Faridbod F., Goldooz H., Badiei A. R., Norouzi P. (2012). A Novel Dichromate-Sensitive
Fluorescent Nano-Chemosensor Using New Functionalized SBA-15. Anal. Chim. Acta.

[30] Zhang J. R., Zeng A. L., Luo H. Q., Li N. B. (2016). Fluorescent
Silver
Nanoclusters for Ultrasensitive Determination of Chromium­(VI) in Aqueous
Solution. J. Hazard. Mater..

[31] Xie J., Wu Z., Sun J., Lv C., Sun Q. (2024). Green Synthesis of
Carbon Quantum Dots Derived from Lycium Barbarum for Effective Fluorescence
Detection of Cr (VI) Sensing. J. Fluoresc..

[32] John A., Roy R. E., Hari H., Zachariah A. K. (2024). Emerging
Stoke’s Shift-Based Cr­(VI) Fingerprint Sensor from Intensely
Blue Fluorescent, High Quantum Yield, Pepitas-Derived Carbon Dots. ACS Applied Optical Materials.

[33] Ye R., Li H., Zhou X. (2024). Facile Preparation
of S, N Co-Doped Carbon Dots and
the Application as an “on-off-on” Fluorescent Sensor
for Detecting Cr­(VI) and Ascorbic Acid/Sulfide. J. Mater. Sci.:Mater. Electron..

[34] Patra S., Golder A. K., Uppaluri R. V. S. (2024). Green Synthesis
of Carbon Dots from
Mature Green Tea Leaves for Label-Free Fluorescence Sensing of Chromium­(VI). Opt. Mater. (Amst)..

[35] Hahn M., Jang M., Cho Y., Bae M., Han M. Y., Piao Y. (2024). Fluorometric Carbon-Dots Nanosensor
for the Detection of Hexavalent
Chromium in Water. Opt. Mater. (Amst)..

[36] Campos B. B., Algarra M., Alonso B., Casado C. M., Jiménez-Jiménez J., Rodríguez-Castellón E., Esteves Da Silva J. C. G. (2015). Fluorescent
Sensor for Cr­(VI) Based in Functionalized Silicon Quantum Dots with
Dendrimers. Talanta.

[37] Ergüder Ö., Şahin Keskin S., Nar I., Trabzon L., Ünlü C. (2022). Aflatoxin B1 Acts as an Effective
Energy Donor to Enhance
Fluorescence of Yellow Emissive Carbon Dots. ACS Omega.

[38] Budak E., Aykut S., Paşaoğlu M. E., Ünlü C. (2020). Microwave
Assisted Synthesis of Boron and Nitrogen Rich Graphitic Quantum Dots
to Enhance Fluorescence of Photosynthetic Pigments. Mater. Today Commun..

[39] Lakowicz J. R., Masters B. R. (2008). Principles of Fluorescence Spectroscopy,
Third Edition. JBO.

[40] Singh V., Kumar V., Yadav U., Srivastava R. K., Singh V. N., Banerjee A., Chakraborty S., Shukla A. K., Misra D. K., Ahuja R., Srivastava A., Saxena P. S. (2017). Sensitive and Selective Detection of Copper Ions Using
Low Cost Nitrogen Doped Carbon Quantum Dots as a Fluorescent Sensing
Plateform. ISSS J. Micro and Smart Systems.

[41] Hutton G. A. M., Martindale B. C. M., Reisner E. (2017). Carbon dots as photosensitisers
for solar-driven catalysis. Chem. Soc. Rev..

[42] Koteswararao D., Bheemudu P., Prameela K. (2025). Microwave-Assisted
Solid-Phase Synthesis
of N-Doped Carbon Dots for the Catalytic Reduction of Malachite Green
and Crystal Violet Dyes. Reaction Kinetics,
Mechanisms and Catalysis.

[43] Wang M., Shi R., Gao M., Zhang K., Deng L., Fu Q., Wang L., Gao D. (2020). Sensitivity Fluorescent Switching
Sensor for Cr (VI) and Ascorbic Acid Detection Based on Orange Peels-Derived
Carbon Dots Modified with EDTA. Food Chem..

[44] Feng Y., Li R., Zhou P., Duan C. (2022). Non-Toxic Carbon Dots Fluorescence
Sensor Based on Chitosan for Sensitive and Selective Detection of
Cr (VI) in Water. Microchemical Journal.

[45] Luo Y., Yuan S., Zhu M., Zhang Z., Cheng B., Xu W., Peng Z. (2025). Poria Cocos-Derived
Carbon Dots for Parallel Detection
of Cr6+/Fe3+ in Complex Environments with Superior Sensitivity. Spectrochim. Acta A Mol. Biomol. Spectrosc..

[46] Wu J., Luo Y., Cui C., Han Q., Peng Z. (2024). Carbon Dots as Multifunctional
Fluorescent Probe for Fe3+ Sensing in Ubiquitous Water Environments
and Living Cells as Well as Lysine Detection via “on-off-on”
Mechanism. Spectrochim. Acta A Mol. Biomol.
Spectrosc..

