# Supplementary material for: Covalently Coupled Phenylenediamine and Citric Acid-Based Carbon Dots: A Dual-Emissive Architecture for Rapid Optical Cr(VI) Sensing and Live-Cell Imaging
Source: ACS Omega. 2026 Jun 25;11(26):38736–49. doi: 10.1021/acsomega.6c01832 (PMC13347337; doi:10.1021/acsomega.6c01832)
Supplement: Supplementary file 1 [file ao6c01832_si_001.pdf]

Supplementary Information

Covalently Coupled Phenylenediamine and Citric Acid-Based Carbon Dots: A Dual-Emissive Architecture for Rapid Optical Cr(VI) Sensing and Live Cell Imaging

Sultan Şahin Keskin[a,b], Canan Çil[c,d], Abdulhalim Kılıç[c,d], Levent Trabzon[b,e], and Caner Ünlü[f]*

a. Department of Genetic and Bioengineering, Alanya Alaaddin Keykubat University, Antalya/Alanya 07425, Turkey

b. MEMS Research Center, Istanbul Technical University, Istanbul 34469, Turkey

c. Department of Molecular Biology and Genetics, Faculty of Science and Letters, Istanbul Technical University, Istanbul 34469, Turkey

d. Molecular Biology-Genetics and Biotechnology Program, MOBGAM, Istanbul Technical University, Istanbul 34469, Turkey

e. Department of Mecanical Engineering, Faculty of Mechanical Engineering, Istanbul Technical University, Istanbul 34437, Turkey

f. Department of Chemistry, Faculty of Science and Letters, Istanbul Technical University, Istanbul 34469, Turkey

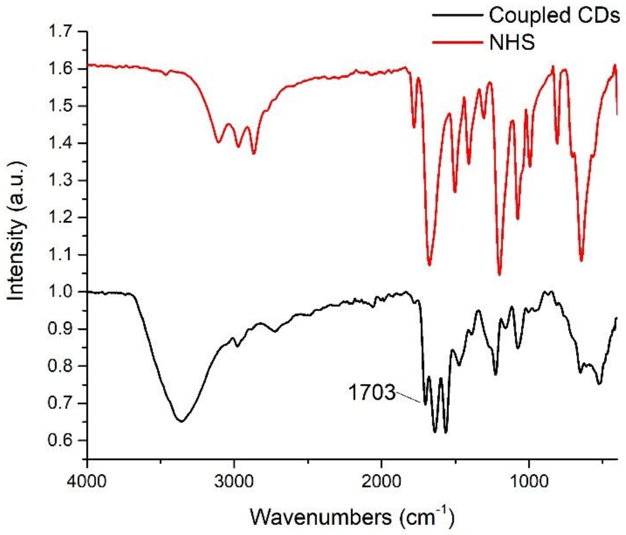

Figure S 1 FT-IR spectra of unprocessed N-Hydroxysuccinimide and coupled CDs

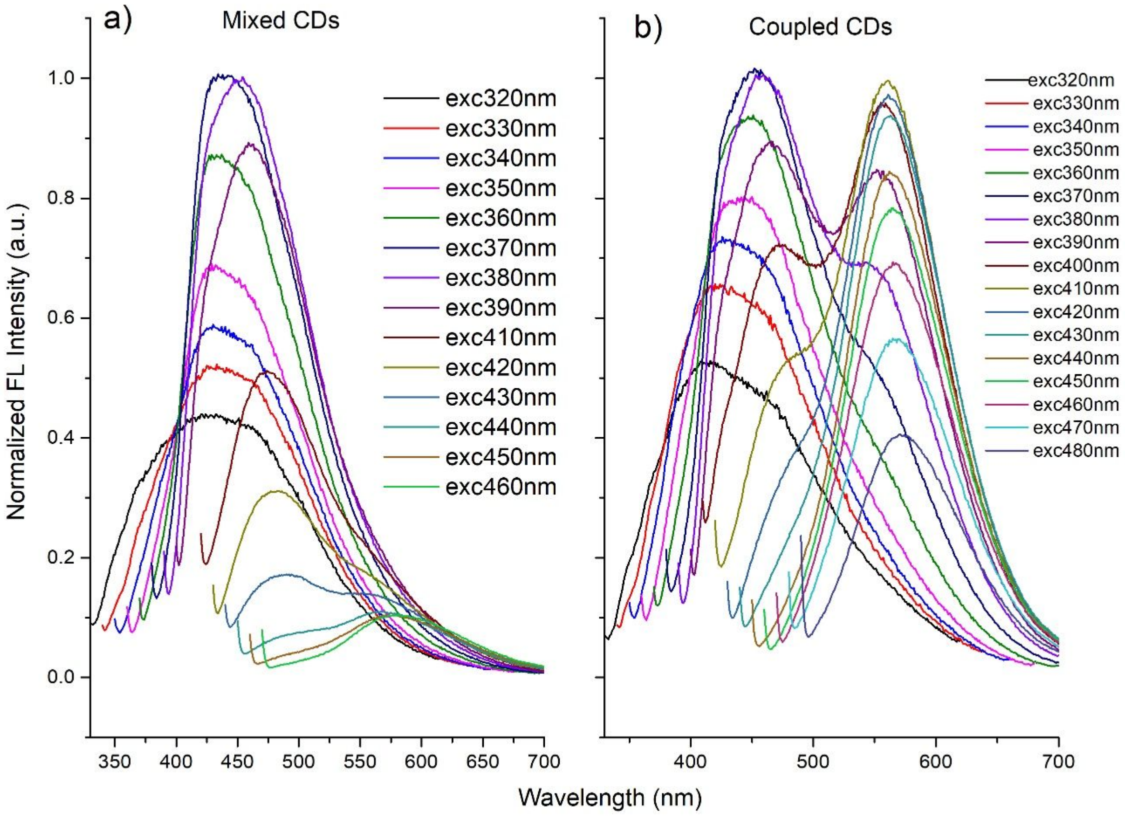

Figure S 2 PL spectra of **a)** mixed CDs with different excitation wavelengths (320-460 nm) and **b)** coupled CDs with different excitation wavelengths (320-480 nm).

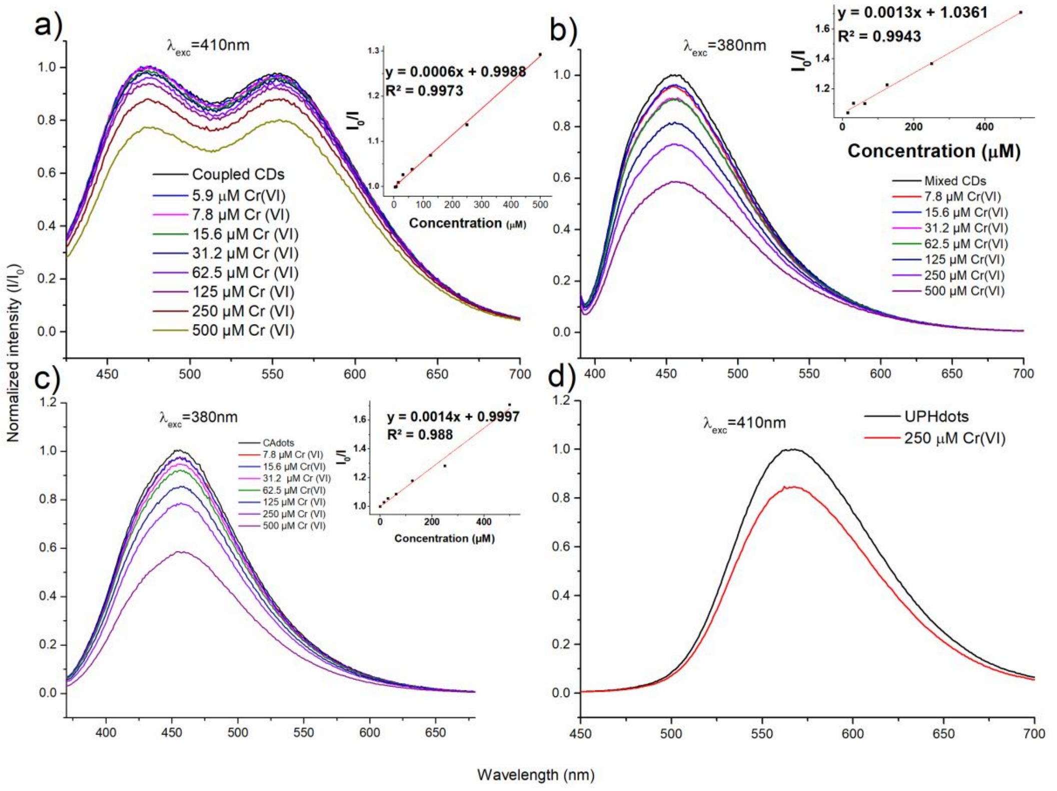

Figure S 3 The fluorescence spectra of **a)** coupled CDs in the presence of different concentrations of Cr(VI) with excitation at 410 nm. Inset of figures a, b, and c represents the linear regression equation of coupled CDs, mixed CDs, and CAdots between the emission intensity ratio of I_0/I versus the concentration of Cr(VI) in the range from 15.6 to 500 μM (**b and c**), and 15.6 to 500 μM (**a**), respectively. **d)** UPHdots quenching efficiency to 250 μM Cr(VI) ions was presented by fluorescent spectra with excitation at 410 nm.

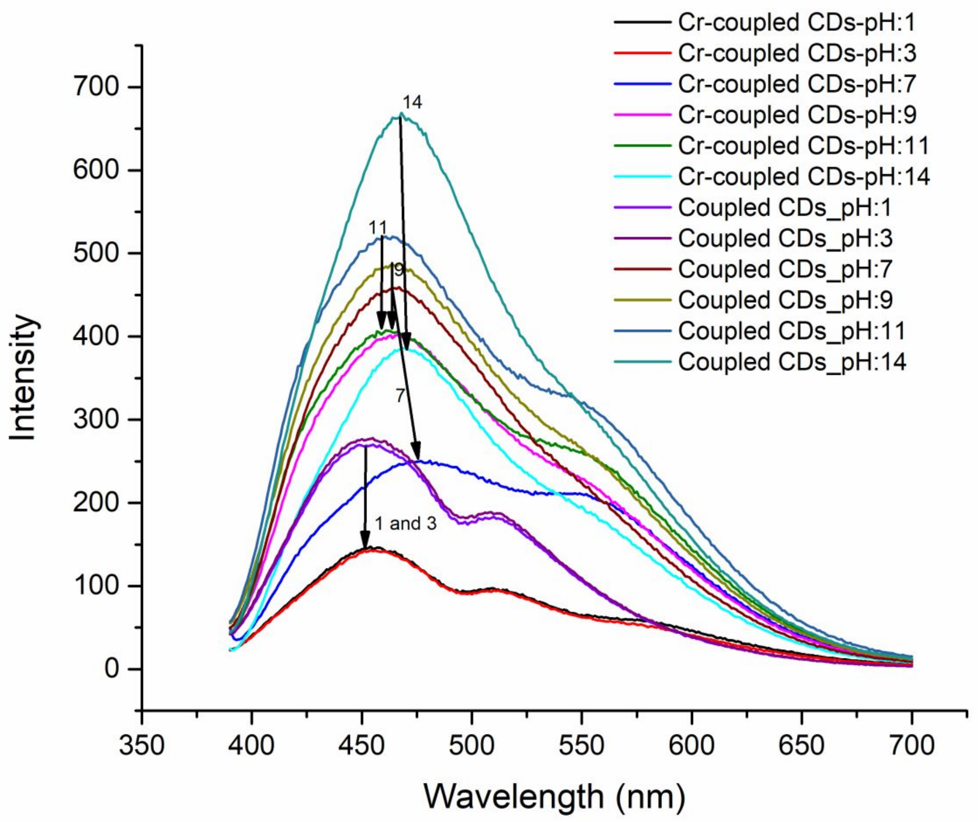

Figure S 4 pH-dependent fluorescence quenching behavior of the coupled CDs toward Cr(VI) (K_2CrO_4) (250 μM) under different pH conditions. All samples were excited at 390 nm.
